# Neonicotinoid seed treatments of soybean provide negligible benefits to US farmers

**DOI:** 10.1038/s41598-019-47442-8

**Published:** 2019-09-09

**Authors:** Spyridon Mourtzinis, Christian H. Krupke, Paul D. Esker, Adam Varenhorst, Nicholas J. Arneson, Carl A. Bradley, Adam M. Byrne, Martin I. Chilvers, Loren J. Giesler, Ames Herbert, Yuba R. Kandel, Maciej J. Kazula, Catherine Hunt, Laura E. Lindsey, Sean Malone, Daren S. Mueller, Seth Naeve, Emerson Nafziger, Dominic D. Reisig, William J. Ross, Devon R. Rossman, Sally Taylor, Shawn P. Conley

**Affiliations:** 1 Department of Agronomy, University of Wisconsin-Madison, Madison, WI 53706 United States; 2 Department of Entomology, Purdue University, West Lafayette, IN 47907 United States; 3 Department of Plant Pathology and Environmental Microbiology, Pennsylvania State University, State College, PA 16801 United States; 4 Department of Agronomy, Horticulture & Plant Science, Brookings, SD 57007 United States; 5 Department of Plant Pathology, University of Nebraska-Lincoln, Lincoln, NE 68583 United States; 6 Department of Plant Pathology, University of Kentucky Research & Education Center, Princeton, KY 42445 United States; 7 Department of Plant, Soil and Microbial Sciences, Michigan State University, East Lansing, MI 48824 United States; 8 Department of Entomology, Virginia Tech Agricultural Research and Extension Center, Suffolk, VA 23437 United States; 9 Department of Plant Pathology and Microbiology, Iowa State University, Ames, IA 50011 United States; 10 Department of Agronomy and Plant Genetics, University of Minnesota, St. Paul, MN 55108 United States; 11 Department of Horticulture and Crop Science, The Ohio State University, Columbus, OH 43210 United States; 12 Department of Crop Sciences, University of Illinois, Urbana, IL 61801 United States; 13 Department of Entomology and Plant Pathology, North Carolina State University, Vernon James Research and Extension Center, Plymouth, NC 27962 United States; 14 Department of Crop, Soil, and Environmental Sciences, University of Arkansas, Little Rock, AR 72204 United States

**Keywords:** Plant sciences, Agroecology

## Abstract

Neonicotinoids are the most widely used insecticides worldwide and are typically deployed as seed treatments (hereafter NST) in many grain and oilseed crops, including soybeans. However, there is a surprising dearth of information regarding NST effectiveness in increasing soybean seed yield, and most published data suggest weak, or inconsistent yield benefit. The US is the key soybean-producing nation worldwide and this work includes soybean yield data from 194 randomized and replicated field studies conducted specifically to evaluate the effect of NSTs on soybean seed yield at sites within 14 states from 2006 through 2017. Here we show that across the principal soybean-growing region of the country, there are negligible and management-specific yield benefits attributed to NSTs. Across the entire region, the maximum observed yield benefits due to fungicide (FST = fungicide seed treatment) + neonicotinoid use (FST + NST) reached 0.13 Mg/ha. Across the entire region, combinations of management practices affected the effectiveness of FST + NST to increase yield but benefits were minimal ranging between 0.01 to 0.22 Mg/ha. Despite widespread use, this practice appears to have little benefit for most of soybean producers; across the entire region, a partial economic analysis further showed inconsistent evidence of a break-even cost of FST or FST + NST. These results demonstrate that the current widespread prophylactic use of NST in the key soybean-producing areas of the US should be re-evaluated by producers and regulators alike.

## Introduction

In the US, the most recent published estimates reflect that approximately 34–44% of planted soybean hectares are treated with neonicotinoid seed treatments (NST)^1^. Based upon inferences drawn from trendlines shown in that work, the current estimate for NST use in soybeans is very likely to exceed 50%. Despite the prevalence of this practice nationally, the work we report here is the first large-scale analysis of soybean yield data. Insecticidal seed treatments of soybean belong to the neonicotinoid class of insecticides that include the active ingredients clothianidin, imidacloprid, and thiamethoxam. Corn and soybean seed treatments represent the largest uses of neonicotinoids nationally, and the higher seeding rate of soybeans mean that they are responsible for higher levels of active ingredient per unit area^2^. It is notable that current NST use rates far exceed historic benchmarks for insecticide use in soybeans; in the decade prior to introduction of neonicotinoid seed treatments, only about 5% of soybean hectares received insecticides^3^. This benchmark reflects that the region where most of US soybeans are grown, the upper Midwest, benefits from a temperate climate and relatively few insect pests, particularly in the early season when NST would provide most crop protection. Recent reviews of insect pest abundance in soybean re-confirm this assessment – early season pests of soybean are still infrequently encountered across the region^4,5^. Soybean aphid, a relatively recent invader to US soybean production, is a notable exception, but it’s abundance and phenology are a poor fit for the early-growing season, when NST are most effective^6,7^.

Recent studies report weak relationships between NST use and effectiveness in preserving crop yield. Specifically for soybean, in a recent multi-environment study in Wisconsin, yield benefit due to the use of insecticide seed treatments was variable^8^. In a comprehensive review conducted by the Environmental Protection Agency - EPA, NSTs were categorized as offering negligible overall benefits to soybean production in the Midwest^9^. A recent multi-state study of management tactics for the key pest in the region, the soybean aphid (*Aphis glycines* Matsumura) demonstrated that crop yield benefits and overall economic returns were marginally affected by NST, while an integrated pest management (IPM) approach, which combined scouting for the pest with foliar insecticide sprays only when the established economic threshold is reached, proved superior in all metrics outlined above^6^. Additionally, other management practices, such as planting date^10^, seeding rates^11^, and possibly region^4^, may affect the seed yield response of insecticide seed treatments. These results suggest that responses are unpredictable and support the notion that prophylactic application of NST across the region may be largely unnecessary. Aside from the fact that a farmer may be incurring unnecessary input costs, a growing body of research suggests that the use of NST in this manner can lead to a host of negative effects upon non-target organisms. It has been reported that neonicotinoids are increasingly detected in terrestrial and aquatic environments^12,13^. Furthermore, studies in the US and elsewhere have evaluated impacts of neonicotinoid on nontarget organisms such as honey bees^13–15^ wild bees^16,17^, monarch butterflies^18^, vertebrates^19,20^, terrestrial and aquatic invertebrates^21,22^ and overall declines in ecosystem function^23^. Although each of these concerns are relevant to the US soybean-producing regions, it is worthwhile to note that key soybean producing states represented in our study include ND, SD, and MN, which rank 3^rd^, 4^th^ and 5^th^, respectively in honey bee colonies ranked by state^24^; many of these are migratory colonies used for pollination of key fruit and nut crops. This presents a key intersection between NST exposure and our principal managed pollinator species with demonstrated sensitivity to this class of compounds.

When implementing an IPM approach, an insecticide is used only when pest populations are expected to reach economically important populations and other management tools are not available or effective^25^. Using NST under an IPM strategy is admittedly challenging due to a lack of secondary pest predictive tools and thus, limited prediction power regarding early season pest populations. Consequently, the approach to NST use in North American annual crops since their introduction in the early 2000’s has been a continent-wide test of an “insurance-based” approach to insect pest management, where the risk of pests across the entire soybean-growing region was assumed to be sufficient to justify the use of insecticide over tens of millions of hectares annually, without corroborating monitoring or real-time yield assessments.

## Results and Discussion

**Figure 1:**
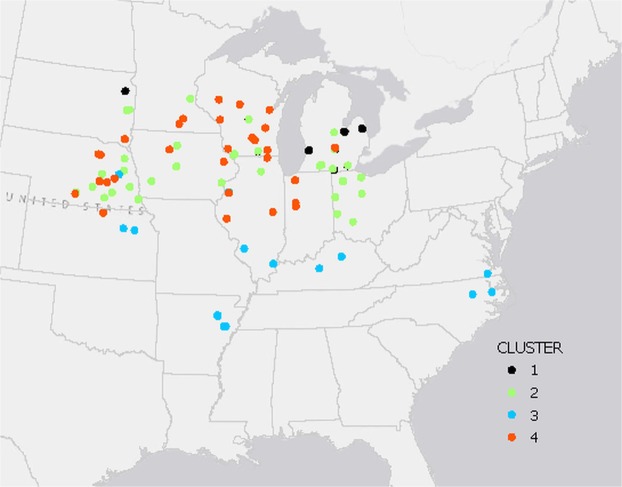
Location of individual experiments that were included in the study. Experiments with the same color belong to a cluster with similar growing environments, as were described in methods and Supplementary Table S1.

For this work, soybean seed yield data from 194 studies were assembled and stratified in four growing environments (Fig. 1) based on soil pH and in-season weather conditions. There were differences in average growing season temperatures and total precipitation among the four clusters (Table S1). The greatest precipitation occurred in locations in clusters 2 and 3. Locations in cluster 4 had the lowest precipitation and greatest average yields were observed in cluster 2.

**Figure 2:**
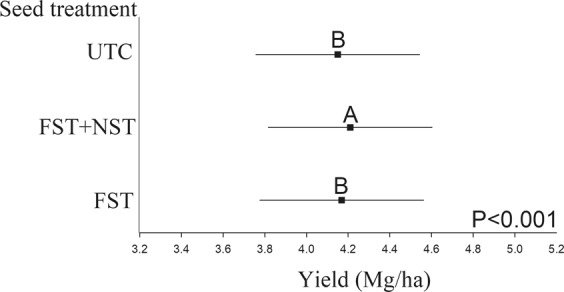
Soybean yield (Mg/ha) due to the applied seed treatments across the entire region. The black rectangles show the mean yield for each treatment and the lines extend to the lower and upper 95% confidence limits. *Note:* FST, fungicide only; FST + NST, fungicide plus neonicotinoid insecticide; UTC, untreated control. Means with the same letter are not significantly different at α = 0.05.

FST, FST + NST, and untreated controls (UTC) were applied in all locations (Table S2). Across the entire region, concurrent use of FST + NST effectively increased soybean yield compared to FST and UTC seed (Fig. 2). Nevertheless, the maximum yield difference compared with fungicide only and from untreated seed was small and reached only 0.04 and 0.06 Mg/ha, respectively. Similar magnitudes of yield differences were observed within clusters 1, 2 and 4 where the effect of seed treatment was significant (Fig. S1).

When repeating the analysis with seed treatments separated by neonicotinoid active ingredients (imidacloprid, thiamethoxam, and clothianidin), across the entire region, concurrent thiamethoxam-based FST + NST resulted in the highest yield (4.3 Mg/ha), and differences compared with the other seed treatments did not exceed 0.13 Mg/ha (Fig. S2). A similar magnitude of yield differences due to seed treatments, separated by neonicotinoid active ingredients, was observed within each cluster (Fig. S3). These results suggest that the yield benefit due to neonicotinoid seed treatments was small but relatively consistent across the entire study area.

**Figure 3:**
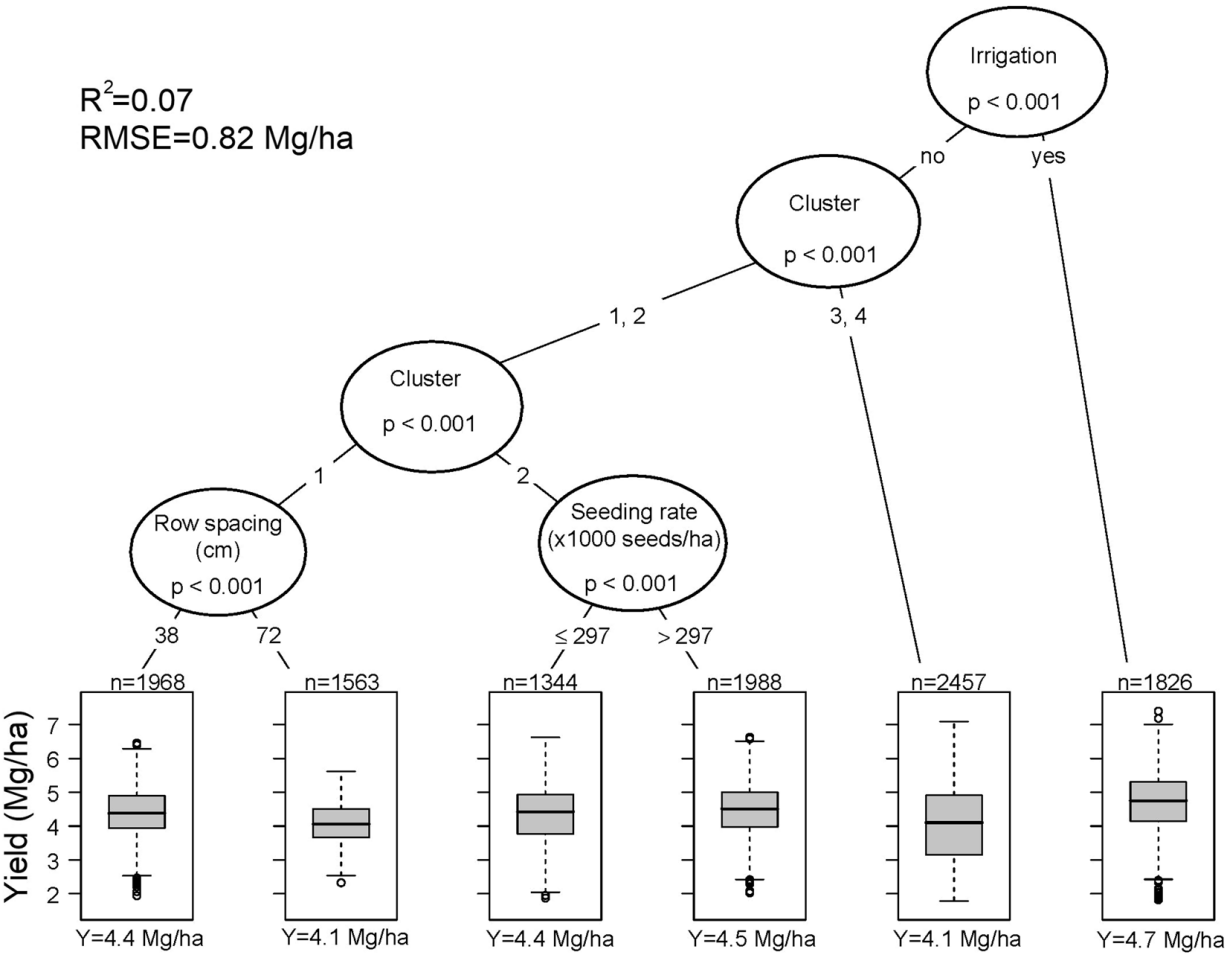
Conditional inference tree for soybean yields (Mg/ha) as affected by environment (clusters) and management practices. In each boxplot, the central rectangle spans the first to the third yield quartiles. The solid line inside the rectangle is the mean which is also numerically shown at the bottom (Y). The number of data points comprising each mean is shown on top of each boxplot (n). The white circles show outlier yields.

Conditional inference tree analysis was used to identify conditional effects of seed treatments with growing environments (clusters) and management practices. Irrigation, followed by cluster, were the most important yield limiting factors followed by the effect of row spacing and seeding rate (Fig. 3). In cluster 1, narrow rows in non-irrigated experiments were associated with yield increase by up to 0.3 Mg/ha whereas in cluster 2, seeding rate greater than 297,000 seeds/ha resulted in the greatest yield.

When repeating the analysis, with treatments separated by neonicotinoid active ingredient (imidacloprid, thiamethoxam, and clothianidin), the results were identical to Fig. 3. This analysis demonstrates that seed treatments, with and without neonicotinoids, have a negligible effect on soybean yield. In all experiments included in this study, the effectiveness of NST was examined concurrently with FST. This is a common practice (51% of treated fields) among farmers across the North-central Midwestern US^26^, and our results suggest that such a practice is unlikely to significantly increase yields across the region. Based on these analyses, we conclude that prophylactic use of seed treatments (with and without neonicotinoids) are not necessary to maximize yield returns across the region, and other management practices that have a more direct impact on soybean seed yield, are much more important considerations^27^, these include sowing date, and cultivar maturity group.

The effectiveness of the combined FST + NST compared with the FST alone appeared to be affected mainly by the use of row spacing, irrigation, and the cultivar’s maturity group (Fig. S4A). The greatest yield benefit of NSTs was observed in 12% of cropping systems that included non-irrigated plants in narrow rows (38 cm) and cultivars with maturity group ≤2 (+0.19 Mg/ha).

Effectiveness of concurrent use of FST + NST compared to the UTC was mainly affected by row spacing and seeding rate (Fig. S4B). The yield benefit in these cropping systems reached 0.22 Mg/ha. It appears that seed treatments including both FST + NST may be more effective in narrow row production systems, measuring 38 cm, and sub-optimal (<198,000 seeds/ha) seeding rates. However, across the Midwestern and North-central US, prevalent row spacings in non-irrigated production systems are 38 cm^27^. Our analysis demonstrates that the optimal management practices are already applied in farmer’s fields, and thus, no additional yield benefit should be expected from FST + NST applications.

Sowing date within a region has a large effect on soybean seed yield^27^. Sowing date (early *vs*. late) has also been reported as a risk factor for pest infestation in various crops and US regions^28^. Early sowing in cold and wet soil can increase the risk for pest infestation and yield reduction^4^. In early and medium sown trials concurrent FST + NST use resulted in 0.06 and 0.1 Mg/ha greater yield than UTC seeds, respectively, whereas no yield benefit was observed in late sown fields across the entire region (Fig. S5). When repeating the analysis by cluster × sowing window, in only half of the early and one fourth of the medium sown clusters FST + NST use resulted in greater yield (between 0.07 to 0.12 Mg/ha) than UTC seeds (Fig. S6).

**Figure 4:**
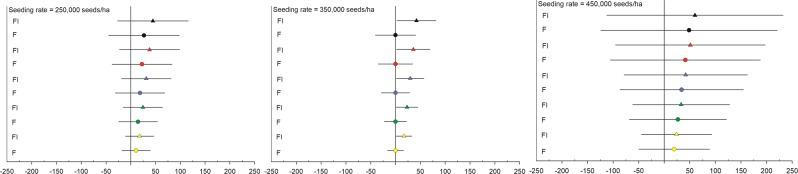
Breakeven cost of fungicide only (F - circles), fungicide + insecticide (FI - triangles) seeds compared to untreated (line at 0 $/ha) for 294 $/Mg (yellow), 404 $/Mg (green), 514 $/Mg (blue), 625 $/Mg (red), and 735 $/Mg (black) soybean price scenarios. The lines extend to the lower and upper 95% confidence limits of each income difference (FST = fungicide − untreated and FST + NST = fungicide + insecticide − untreated seed).

The results in our study show an environmental- and management-specific soybean seed yield response due to the use of neonicotinoid seed treatments. In general terms, these small yield benefits call into question the economic return on investment of prophylactic applications of neonicotinoid seed treatments. Partial economic analysis of the observed yield increases under 294, 404, 514, 625, and 735 $/Mg soybean price scenarios at 250,000, 350,000, and 450,000 seeds/ha seeding rates showed that both FST and FST + NST seed should not cost more than UTC seed (Fig. 4). For all price scenarios, breakeven cost of FST + NST was significant only at 350,000 seeds/ha (P = 0.034). Hypothetically, a higher treatment cost would be justified if soybean prices were greater than 735 $/Mg. However, this is extremely unlikely; the average monthly soybean price during the last 10 years was 472 $/Mg and the maximum price has never reached this threshold (maximum of 684 $/Mg in Aug 2012, a year when drought impacted many states in the soybean-growing region)^29^.

The lack of consistent yield benefits attributable to NST, coupled with mounting reports of potential environmental risks, highlight that the current default approach of prophylactic applications of NST in soybeans in the US should be re-evaluated. Adjusting other soybean management practices, such as sowing date, row spacing and seeding rate, appear to have a greater potential to increase soybean yields across the entire examined region compared to neonicotinoid use^26,27^. Such practices represent a net-zero environmental burden compared with the prophylactic use of neonicotinoid treated seeds. IPM, a decision-making process based on scientific data to identify and reduce both yield limiting risks from pests and from pest-management related strategies, can be followed as an alternative to some extent. Although sporadic yield limiting pests are difficult to predict in advance, our data demonstrate that yield-limiting populations of these pests are uncommon across our growing region and that current use rates of NST are likely to far outpace their utility for soybean pest management. This observation is supported by previous analyses^9^, and by recent reviews of the prevalence and population dynamics of soybean insect pests across the region^4,5^.

In response to heightened concerns about the non-target effects of NST use in annual crops, the European Commission restricted the use of clothianidin, imidacloprid and thiamethoxam neonicotinoid insecticides. Initially a moratorium, these restrictions were renewed in 2018 and expanded to a complete ban on all outdoor uses of the compounds in 2018^30^. The restriction in neonicotinoids initially resulted in the use of alternative insecticide seed treatments or foliar applications by farmers^31^. Thus, an important issue is the anticipated response of farmers to a similar use restriction in the US. Such action may lead to increased use of alternative treatments and products which in turn, can result in different environmental issues. Our data provide some measure of security for soybean producers and other agricultural professionals that pest pressure is low across the key soybean-growing regions of the US. This provides empirical data for researchers, regulators, and seed sales staff to inform producers about the likelihood of measurable pest management and yield benefits associated with NST use, and we can infer that other, similar pest management approaches would be equally unnecessary in the absence of pests. This affords the industry an opportunity for a correction where NST use rates align more closely with pest incidence and risk factors. Given the demonstrable non-target issues associated with the current approach to NST use, we argue that this correction is not only advisable, but necessary if these pest management tools are to be preserved for occasions where they can provide benefit.

## Conclusions

Our analysis, spanning 12 years and 14 soybean-producing states, provides no empirical support for continuing the current approach of blanket NST use in soybeans. On the contrary, our data suggest that this approach provides little to zero net benefit in most cases, and that meaningful (i.e. significant) gains are likely to be realized by site-specific management practices, independent of NST use. Although we do not have site-specific pest data to identify the mechanisms behind our lack of observed pest management benefits, our results are given context by historical data that reflect the scarcity of soybean pests targeted by this approach. This means that throughout most soybean-producing regions of the US, the period of pest protection provided by NSTs does not align with economically significant pest populations. Absent economic infestations of pests, there is no opportunity for this plant protection strategy to provide benefit to most producers.

## Methods

Soybean seed yield data from 194 randomized and replicated field studies, which were conducted specifically to evaluate the effect of seed treatments on soybean seed yield at sites within each of 14 states from 2006 through 2017, were assembled for this study. To include an individual experiment in the combined database, presence of replicated and randomized neonicotinoid seed treatments was essential. The final database included 11,146 plot-specific soybean seed yields. For all experiments, soil pH and information about nine major management practices, including irrigation, sowing date, cultivar maturity group, tillage operations, previous year crop, row spacing, seeding rate, double crop system, and manure application were recorded.

For each study, weather data were obtained from the PRISM dataset^32^. Weather variables included daily minimum and maximum temperatures (Tmin and Tmax, respectively), and precipitation. In all studies, sowing date as day of year was set to zero and the 30-d specific weather conditions, beginning 30-d before sowing (DBS) up to 150 days after sowing (DAS), and season-wide (0–150 DAS) were calculated.

Since individual experiments were located in different regions, the effect of environment on soybean yield was assumed to be significant. Therefore, to account for non-management-related effects on yield, cluster analysis was used to stratify experiments into similar growing environments based on soil pH and the aforementioned weather variables. Additionally, to enhance the clustering model, cumulative precipitation from sowing to 60 DAS, from sowing to 90 DAS, from sowing to 120 DAS, and use of irrigation were included as independent variables. Finally, coordinates (latitude and longitude), and sowing date were also included in the model to capture additional non-weather-related information (e.g., photoperiod). Variables were then standardized to mean = 0 and standard deviation = 1 and clusters were created using PROC FASTCLUS in SAS 9.4 (SAS Institute Inc., 2016). In this method, the iterative algorithm minimizes the sum of squared distances from the cluster means. The clustering is done using Euclidean distances computed from numeric variables. This kind of clustering method is often called a k-means model, since the cluster centers are the means of the observations assigned to each cluster. In each iteration, the least-squares criterion is reduced until convergence is achieved. We used adaptive training by using the DRIFT option in which the cluster seed is updated as the current mean of the cluster each time an observation is added. We specified LEAST = 2 which minimizes the root mean square difference between the data and the corresponding cluster means. Using canonical analysis, visual evaluation of the clusters showed a high degree of separation and small overlap among the clusters (Fig. S7).

In the first analysis, a multilevel model, as described previously^33^, was used in PROC MIXED in SAS 9.4 to quantify the effect of seed treatments on soybean seed yield across clusters and the nine major aforementioned management practices. The seed treatment variable was used as a fixed effect and included three levels: fungicide only (FST), fungicide + neonicotinoid insecticide (FST + NST), and untreated control (UTC). The stratification of environments and management practices were introduced in the model as random effects. For management practices, continuous variables (sowing date, seeding rate, cultivar maturity group, and row space) were transformed to categorical. Sowing date and seeding rate had three levels, (early, medium, and late sowed fields and sparse, medium, and dense sowed, respectively) that corresponded to three percentiles (<30^th^, 30^th^ to 70^th^, and >70^th^). The median was used to create two levels for maturity group whereas for row spacing, two spacings were used across all studies (38 and 76 cm). Then, a combined management practice variable (mgm) was created as a nine-way combination of the levels of the nine recorded management practices. Thus, random effects included clusters, experiments within clusters, replication nested within experiments and clusters, the seed treatment nested within replications, mgm, the seed treatment nested within mgm, and the seed treatment nested within mgm and clusters. Degrees of freedom were calculated using the Kenward-Rodger approximation, 95% confidence limits were calculated, and the adjdfe = row and adjust = simulate adjustments were used for multiple pairwise means comparisons at 5% confidence level. These adjustments were used to take into account the fact that observations are not equal at all levels of the independent variables and thus, degrees of freedom are subject to variability.

When the analysis was repeated for seed treatments separated by active ingredient, the fixed effect variable had six levels: fungicide only, fungicide plus thiamethoxam (application rate range: 0.0756 to 0.1512 mg. a.i. per seed.), fungicide plus imidacloprid (application rate range: 0.10 to 0.2336 mg. a.i. per seed), fungicide plus clothianidin (application rate range: 0.081 to 0.13 mg. a.i. per seed), fungicide plus both imidacloprid and clothianidin (application rates: 0.1013 mg a.i. per seed and 0.1056 mg a.i. per seed, respectively), and an untreated control (UTC). All fungicides were applied according to labeled rates. The aforementioned model was used for this analysis.

To assess the seed treatment-related yield differences within each cluster, random effects included experiments, replication nested within experiments, the seed treatment nested within replications, mgm, and the seed treatment nested within mgm. The same model was also used when the analysis was repeated for seed treatments separated by active ingredient.

In our study, the levels of the nine major management practices were not replicated or randomized across all individual experiments. Therefore, evaluation of the levels of individual management practices with the greatest effect on seed treatment performance using traditional linear models (e.g., analysis of variance, multiple linear regression) are not appropriate. Consequently, we used the conditional inference regression trees methodology, within the “partykit” package in R (R development Core team, 2016). To evaluate which management practices had the greatest impact on the effectiveness of insecticide only and FST + NST, in every individual experiment (n = 194) and for every nine-way management combination, we calculated the following respective yield differences: FST + NST minus FST and FST + NST minus UTC. In these analyses, the criterion for the independence test was based on a univariate p-value of alpha = 0.01. Additionally, to ensure adequate power at all steps, each intermediate node had to account for >25% of total observations and a terminal node had to consist of >10% of total observations. To avoid overfitting and enhance interpretability, the maximum tree depth was set to 5 nodes.

For the partial economic analysis, yield in every plot in every trial with seeding rate of 250,000, 350,000, and 450,000 seeds/ha were converted to profit for five soybean price scenarios: 294, 404, 514, 625, and 735 $/Mg. Then, the profit for each scenario (seeding rate × seed treatment (FST, FST + NST, UTC) × soybean price) was used as dependent variable in a mixed model analysis as was described earlier.

## Supplementary information

Supplemental information
