# Supplementary material for: Neonicotinoid seed treatments of soybean provide negligible benefits to US farmers
Source: Sci Rep. 2019 Sep 9;9:11207. doi: 10.1038/s41598-019-47442-8 (PMC6733863; doi:10.1038/s41598-019-47442-8)
Supplement: Supplementary file 1 — Supplemental information [file 41598_2019_47442_MOESM1_ESM.docx]

**Neonicotinoid seed treatments of soybean provide negligible benefits to US farmers**

Spyridon Mourtzinis^1^*, Christian H. Krupke^2^, Paul D. Esker^3^, Adam Varenhorst^4^, Nicholas J. Arneson^5^, Carl A. Bradley^6^, Adam M. Byrne^7^, Martin I. Chilvers^7^, Loren J. Giesler^5^, Ames Herbert^8^, Yuba R. Kandel^9^, Maciej J. Kazula^10^, Catherine Hunt^9^, Laura E. Lindsey^11^, Sean Malone^8^, Daren S. Mueller^9^, Seth Naeve^10^, Emerson Nafziger^12^, Dominic D. Reisig^13^, Jeremy Ross^14^, Devon R. Rossman^7^, Sally Taylor^8^, and Shawn P. Conley^1^

^1^Department of Agronomy, University of Wisconsin-Madison, Madison WI 53706, United States.

^2^Department of Entomology, Purdue University, West Lafayette, IN 47907 United States.

^3^Department of Plant Pathology and Environmental Microbiology, Pennsylvania State University, University Park, PA 16802, United States.

^4^Department of Agronomy, Horticulture & Plant Science, Brookings, SD 57007, United States.

^5^Department of Plant Pathology, University of Nebraska-Lincoln, Lincoln, NE 68583, United States.

^6^Department of Plant Pathology, University of Kentucky Research & Education Center, Princeton KY, 42445-0469, United States.

^7^Department of Plant, Soil and Microbial Sciences, Michigan State University, East Lansing, MI 48824, United States.

^8^Department of Entomology, Virginia Tech Agricultural Research and Extension Center, Suffolk, VA 23437, United States.

^9^Department of Plant Pathology and Microbiology, Iowa State University, Ames, IA 50011, United States.

^10^Department of Agronomy and Plant Genetics, University of Minnesota, St. Paul, MN 55108, United States.

^11^Department of Horticulture and Crop Science, The Ohio State University, Columbus, OH 43210, United States.

^12^Department of Crop Sciences, University of Illinois, Urbana, IL, 61801, United States.

^13^North Carolina State University Vernon James Research and Extension Center Plymouth, NC 27889, United States.

^14^Department of Crop, Soil, and Environmental Sciences, University of Arkansas, Little Rock, AR, 72204, United States.

*Correspondence to agstat001@gmail.com

**Table S1. Average soil pH, seasonal minimum (Tmin) and maximum (Tmax) temperatures and precipitation, and soybean yield within each cluster.**

| Cluster | Variable | Mean | Standard Deviation |
| --- | --- | --- | --- |
| 1 | pH | 6.7 | 0.6 |
|  | average season Tmin (˚C) | 13.4 | 0.9 |
|  | average season Tmax (˚C) | 25.5 | 0.8 |
|  | cumulative season precipitation (mm) | 356 | 53 |
|  | Yield (Mg/ha) | 4.4 | 0.7 |
| 2 | pH | 6.5 | 0.6 |
|  | average season Tmin (˚C) | 14.3 | 1.2 |
|  | average season Tmax (˚C) | 26.1 | 0.9 |
|  | cumulative season precipitation (mm) | 479 | 55 |
|  | Yield (Mg/ha) | 4.5 | 0.8 |
| 3 | pH | 6.6 | 0.4 |
|  | average season Tmin (˚C) | 18.8 | 1.7 |
|  | average season Tmax (˚C) | 30.4 | 1.4 |
|  | cumulative season precipitation (mm) | 436 | 150 |
|  | Yield (Mg/ha) | 4.3 | 1.1 |
| 4 | pH | 6.7 | 0.5 |
|  | average season Tmin (˚C) | 14.8 | 0.8 |
|  | average season Tmax (˚C) | 28.1 | 0.8 |
|  | cumulative season precipitation (mm) | 259 | 66 |
|  | Yield (Mg/ha) | 4.1 | 1.1 |

**Table S2. Number of observations of each seed treatment within each cluster.**

| Cluster | Seed treatment | Number of observations |
| --- | --- | --- |
| 1 | Untreated | 1111 |
|  | Fungicide + Insecticide | 1471 |
|  | Fungicide | 1356 |
| 2 | Untreated | 1263 |
|  | Fungicide + Insecticide | 1597 |
|  | Fungicide | 1455 |
| 3 | Untreated | 181 |
|  | Fungicide + Insecticide | 248 |
|  | Fungicide | 192 |
| 4 | Untreated | 572 |
|  | Fungicide + Insecticide | 920 |
|  | Fungicide | 780 |


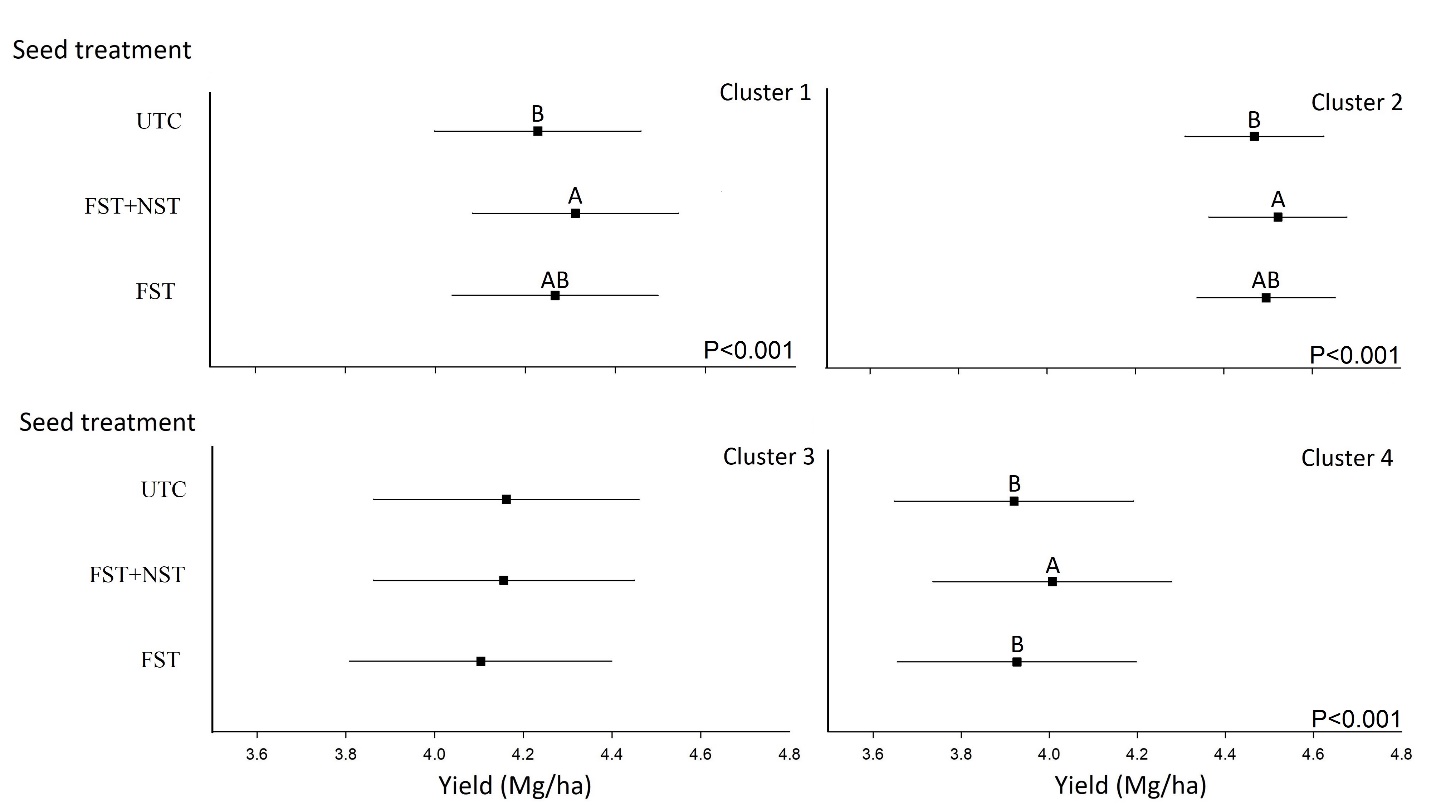


**Figure S1. Soybean yield (Mg/ha) due to the applied seed treatments within each cluster.** The black rectangles show the mean yield for each treatment and the lines extend to the lower and upper 95% confidence limits. *Note:* FST, fungicide only; FST+NST, fungicide plus insecticide; UTC, untreated control. Means with the same letter are not significantly different at α=0.05.


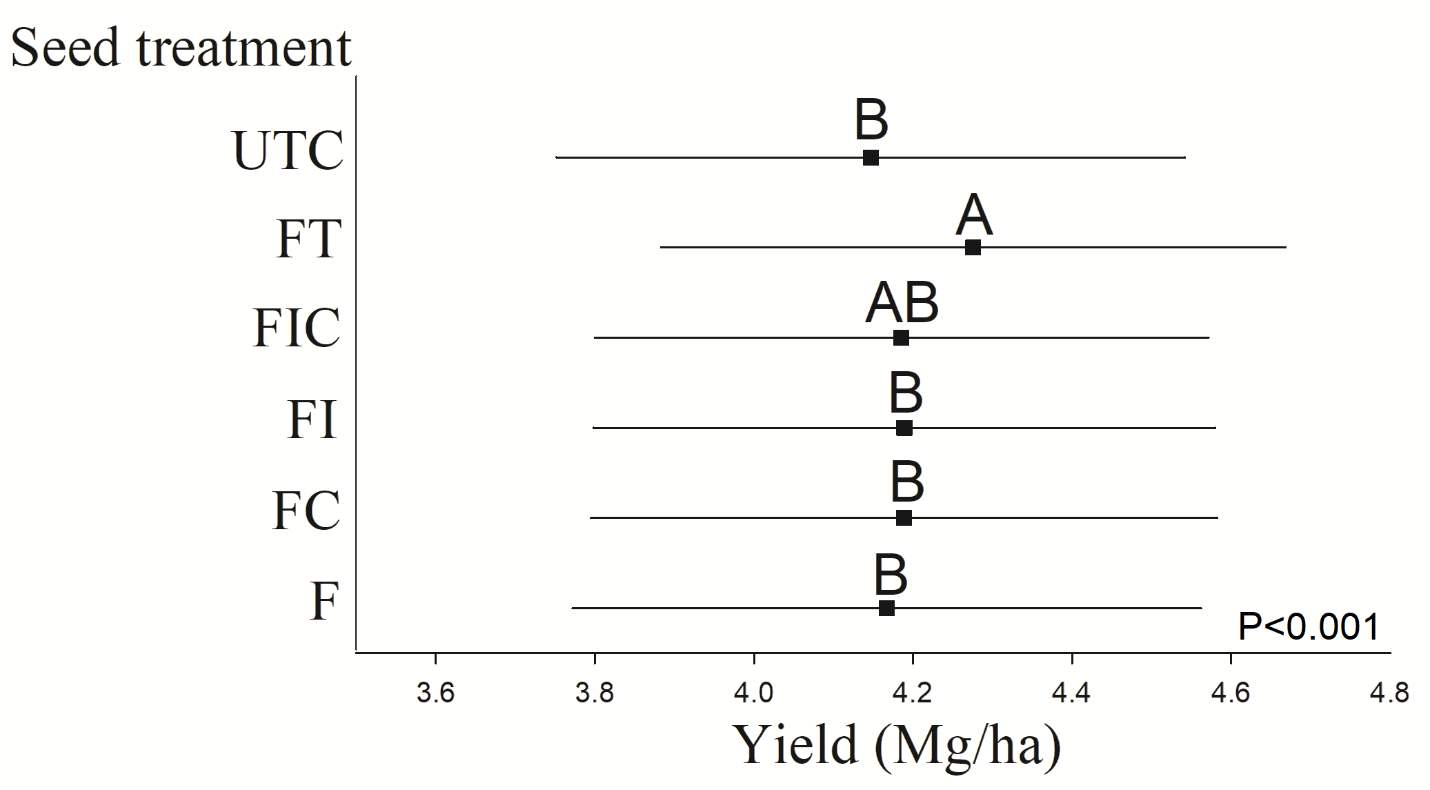


**Figure S2. Soybean yield (Mg/ha) due to the applied seed treatments across the entire region.** The black rectangles show the mean yield for each treatment and the lines extend to the lower and upper 95% confidence limits. *Note:* F, fungicide only; FT, fungicide plus insecticide (thiamethoxam); FI, fungicide plus insecticide (imidacloprid); FC, fungicide plus insecticide (clothianidin); FIC, fungicide plus insecticide (imidacloprid + clothianidin); UTC, untreated control. Means with the same letter are not significantly different at α=0.05.


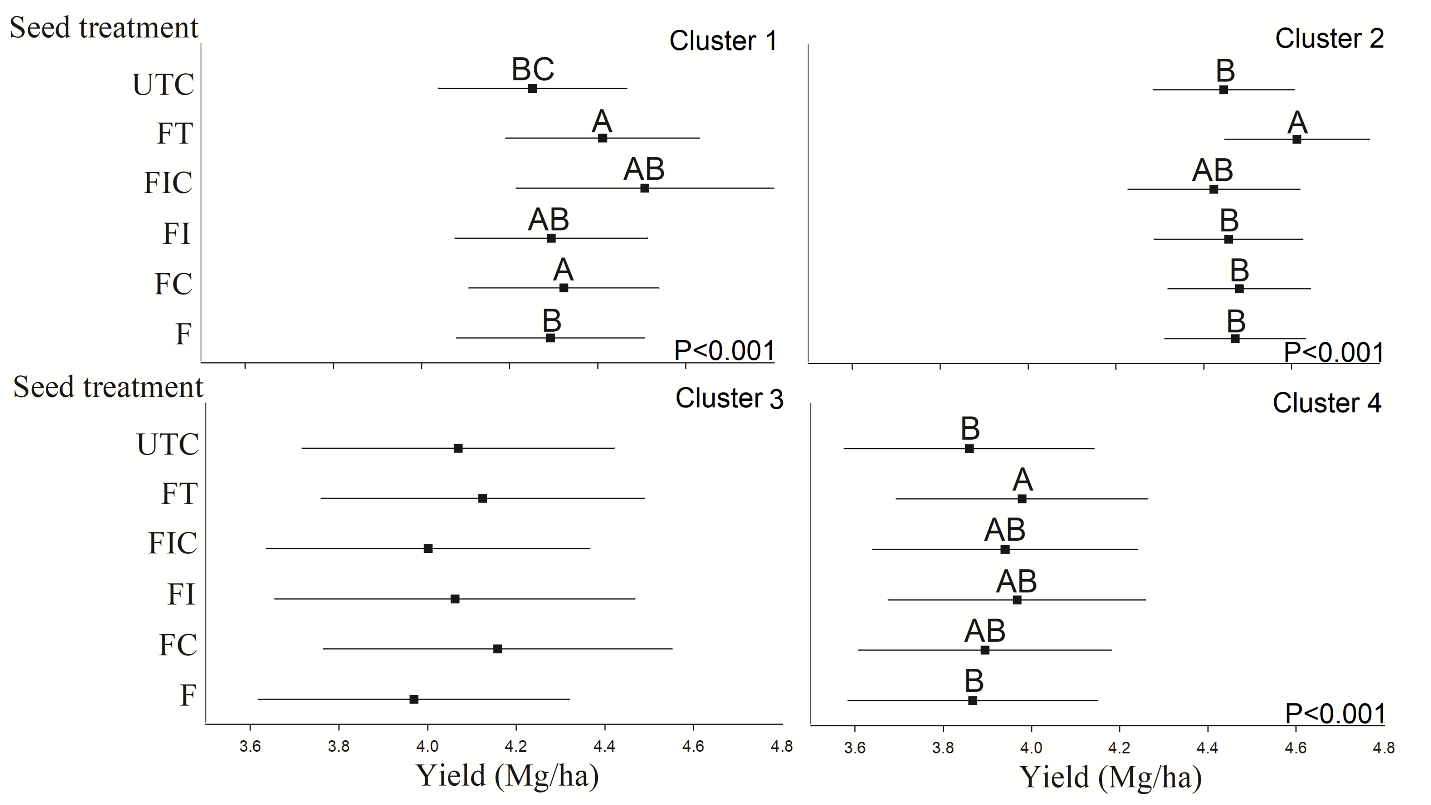


**Figure S3. Soybean yield (Mg/ha) due to the applied seed treatments within each cluster.** The black rectangles show the mean yield for each treatment and the lines extend to the lower and upper 95% confidence limits. *Note:* F, fungicide only; FT, fungicide plus insecticide (thiamethoxam); FI, fungicide plus insecticide (imidacloprid); FC, fungicide plus insecticide (clothianidin); FIC, fungicide plus insecticide (imidacloprid + clothianidin); UTC, untreated control. Means with the same letter are not significantly different at α=0.05.


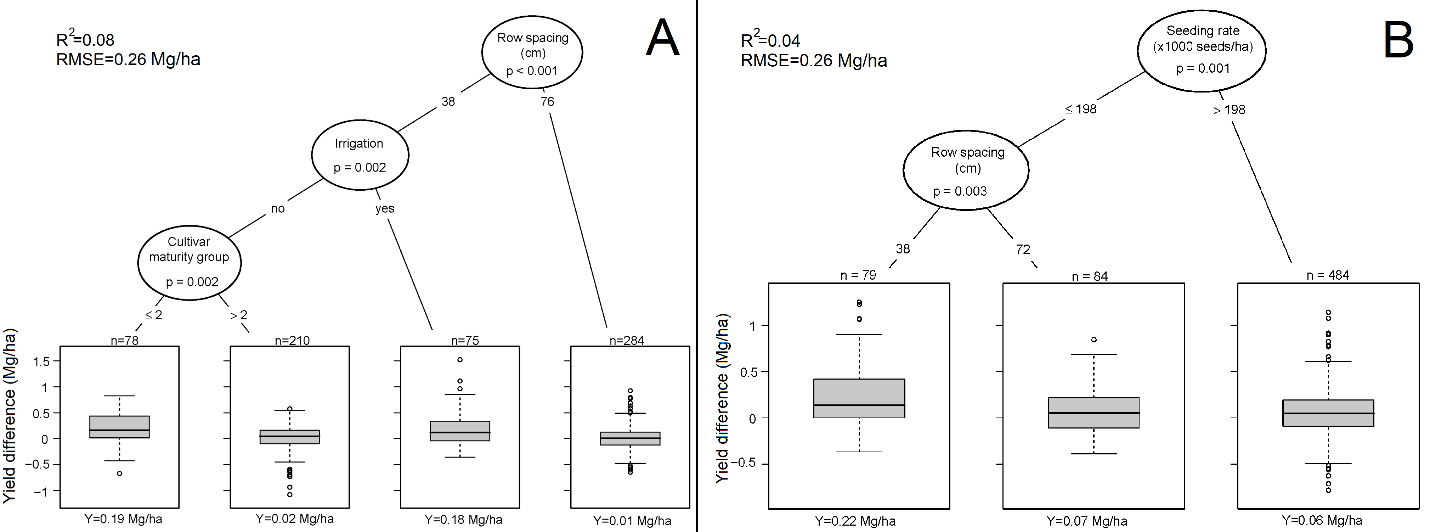


**Figure S4. Conditional inference trees for A) yield difference between fungicide + insecticide- vs. fungicide-treated soybean yields (Mg/ha), and B) yield difference between fungicide + insecticide-treated vs. untreated soybean yields (Mg/ha) as affected by environment (clusters) and management practices.** In each boxplot, the central rectangle spans the first to the third yield quartiles. The solid line inside the rectangle is the mean which is also numerically shown at the bottom (Y). The number of yields is shown on top of each boxplot (n). The white circles show outlier yields.


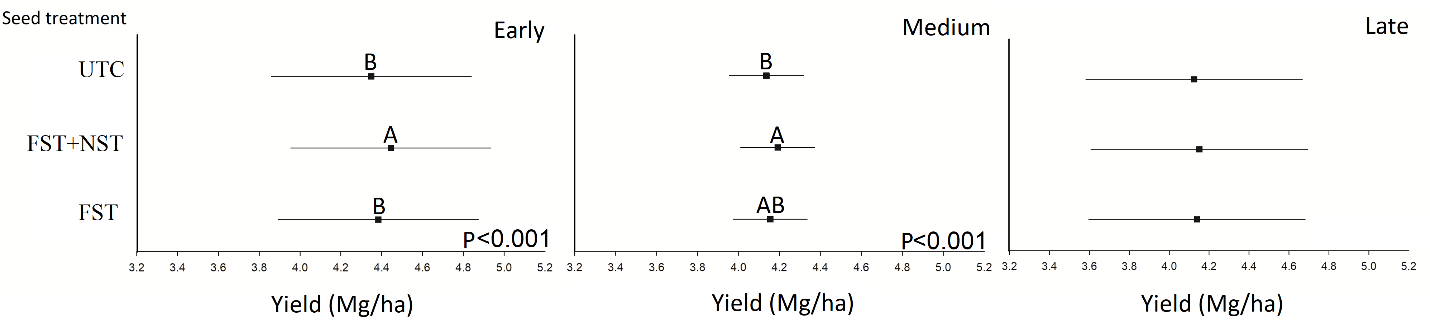


**Figure S5. Soybean yield (Mg/ha) due to the applied seed treatments across the entire region in early, medium, and late sowed trials.** The black rectangles show the mean yield for each treatment and the lines extend to the lower and upper 95% confidence limits. *Note:* FST, fungicide only; FST+NST, fungicide plus insecticide; UTC, untreated control. Means with the same letter are not significantly different at α=0.05.


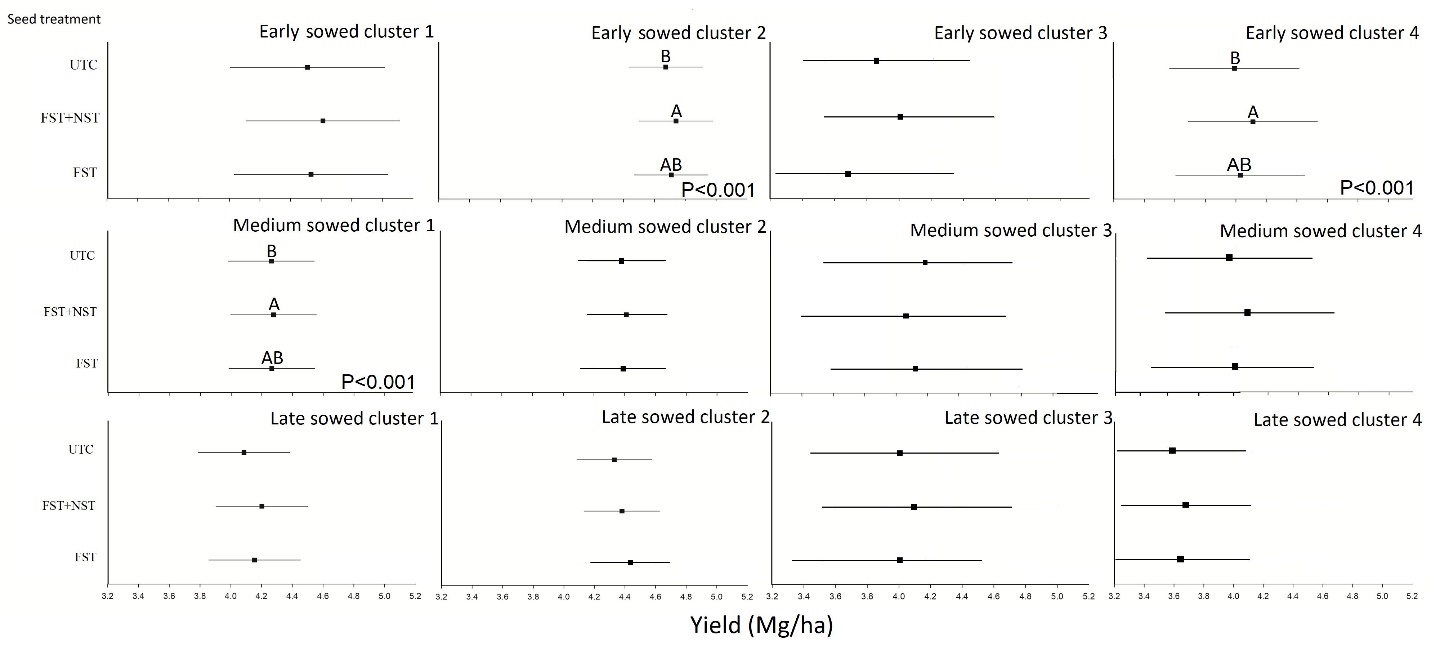


**Figure S6. Soybean yield (Mg/ha) due to the applied seed treatments in early, medium, and late sowed trials in every cluster.** The black rectangles show the mean yield for each treatment and the lines extend to the lower and upper 95% confidence limits. *Note:* FST, fungicide only; FST+NST, fungicide plus insecticide; UTC, untreated control. Means with the same letter are not significantly different at α=0.05.


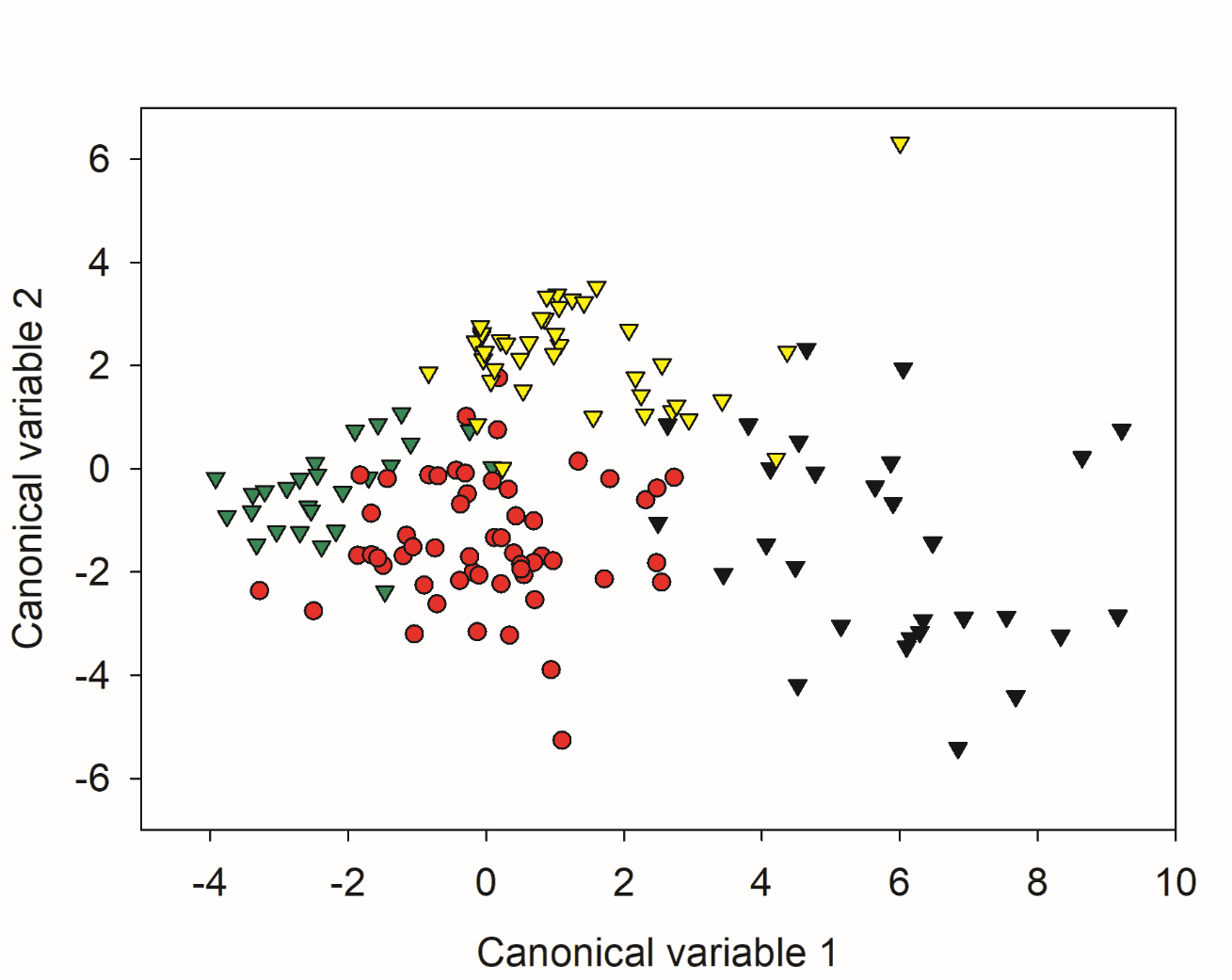


**Figure S7.** Visual separation of the four clusters based on two canonical variables. The canonical variables were created using canonical discriminant analysis of the soil and weather variables, for details please see materials and methods.
